# Hormone Receptor Positive Breast Cancer in Young Women: A Review

**DOI:** 10.1002/jso.27963

**Published:** 2024-10-29

**Authors:** Nicola McShane, Alexandra Zaborowski, Mary O'Reilly, Damian McCartan, Ruth Prichard

**Affiliations:** ^1^ St Vincent's University Hospital Dublin Ireland

## Abstract

The global incidence of hormone‐positive breast cancer (HR+ BC) in young women is rising, though the underlying reasons remain unclear. HR+ disease in younger women appears to represent a distinct clinical entity compared to that in older women, exhibiting distinct clinicopathological characteristics, outcomes and responses to treatment. Despite these differences, there is a paucity of large‐volume data focusing on young women with HR+ in contemporary literature. Hormone receptor positive breast cancer in young women is associated with poorer prognoses compared to older women. Additionally, early age onset breast cancer presents unique challenges, including concerns related to fertility, the toxic effects of therapeutic agents, and specific surgical considerations. The purpose of this review is to report the existing literature on HR+ disease in young women.

## 1 Introduction

Breast cancer (BC) represents the most prevalent malignancy and leading cause of cancer‐related death in women age < 40 years [1]. While comprising only a small proportion of all BC cases, registry‐based studies demonstrate an average increase in incidence of 0.7%–2.6% annually worldwide [2, 3, 4].

Tumours in younger patients display different clinical and oncological features to that of older women [5]. Hormone receptor positive (HR+) BC (defined as the expression of the oestrogen receptor (ER) or progesterone receptor (PR) or both) represents the most common subtype in younger patients, accounting for approximately two‐thirds of all BC cases [6].

Population‐based studies have consistently shown that younger women are more likely to present with stage IV disease with aggressive features compared to their older counterparts [7, 8], and are more likely to die as a result of their cancer [9, 10, 11]. This is particularly true in HR+ BC, with premenopausal women with HR+ HER2− BC experiencing higher rates of recurrence and death [12, 13].

The definition of young women in breast oncology is not standardised, with discordance amongst publications. Age < 40 will be used as the descriptive in accordance with the European Society of Medical Oncology (ESMO) [14], unless otherwise stated within the review. The purpose of this article is to deliver a comprehensive analysis of hormone‐positive BC in young women, emphasising the aetiological background, current therapeutic approaches and specific considerations pertinent to this patient cohort.

## 2 Epidemiology

BC is the most common cancer in women, with approximately 2.3 million new cases diagnosed annually worldwide [15]. The incidence amongst younger women has shown a gradual increase in recent decades. Amongst European women < 40 years an average annual increase of 1.2% per year was observed between 1990 and 2008 [16]. This was twice as high in those age 15–34 (+2.0%) compared to those age 35–39 (+1.1%). A similar increase was observed in the United States, where a study of the SEER database from 1992 to 2009 showed an annual increase of 8% in women aged 25–39 years with HR+ disease [17]. Australia recorded an average annual increase of 1.39% in women age 25–49 from 1982 to 2018 [18]. Quantification of incidence in Asia is not as well established, however, in China age standardised percentage of BC in those age < 35years increased from 4.0% in the early 2000s to 5.9% in 2017. The median age of BC in Asian women is significantly lower than across other continents, at 45–49 years in East Asian countries [19, 20] compared to 62–64 years in the USA.

HR+ disease is the most common subtype across all ethnicities, however, the highest rates of Luminal A (HR+ HER2−) and Luminal B (HR+ HER2+) disease occur amongst Caucasian women when compared to Black, Asian or pacific islander and Hispanic women. The higher proportion of HR+ disease amongst Caucasian women is most significant in those age < 35 [21].

Certain risk factors for young HR+ BC differ from those associated with older patients. Multiparity is known to be protective in post‐menopausal women, however, is associated with a greater risk of disease development in younger women. Similarly, obesity is associated with an increased risk of BC in post‐menopausal women whilst risk of HR+ BC in premenopausal women is inversely related to BMI [22]. The strong association between increased BMI and HR+ BC may suggest that the hormonal changes seen in overweight women such as lower estradiol, sex‐binding hormone and progesterone levels and higher free testosterone levels [23] may play a protective role against tumorigenesis (Table 1).

**Table 1:** Frequent genetic mutations observed in young HR + BC.

Gene	Mutation/deletion in premenopausal women (%)
PIK3CA	43
GATA3	17
CCND1	14
BRCA2	11
MAP3K1	10
FGFR1	10
BRCA1	9
TP53	5

*Note:* Genomic driver alterations observed in HR + HER2− BC in pre‐menopausal women from SOFT study (*n* = 1276). Data derived from Luen et al. [24].

## 3 Genetics

Genetic variants in cancer susceptibility genes account for approximately 10% of all cases of BC. While several germline mutations predisposing to early onset BC have been identified, no single genetic mutation has been recognised as predominantly significant. Genomic sequencing in patients from the Young Women's Breast Cancer Study identified higher rates of GATA3 and ARID1A mutations in women < 35 with luminal A cancer compared to those > 45 (43% vs. 12% and 18% vs. 2%, respectively) [25]. The influence of GATA3 in BC is undetermined, however, it is suggested that it carries poor prognosis [26]. Whole transciptome profiling of 187 pre‐menopausal patients with BC found that GATA3 was exclusively mutated in HR+ subtypes [27].

In young women with Luminal A ductal carcinomas, lower rates of PIK3CA mutations (14% vs. 38%) were observed, which may in part explain the poor outcomes associated with these tumours [28]. PIK3CA mutations exist in approximately 26% of BC overall, and are associated with favourable characteristics, such as lower tumour grade and decreased rates of lymph node metastases [29].

TP53 is the most commonly mutated gene in cancer. The tumour antigen p53 protein acts as a checkpoint following DNA damage. Women harbouring a TP53 pathogenic variant carry a lifetime BC risk of up 90%, higher than that of BRCA carriers. While HR+ BC is less commonly associated with TP53 mutations, they are the only subtype of BC in whom TP53 mutations are associated with inferior outcomes. This is significant only amongst those with Luminal B subtype [30].

## 4 Clinical and Molecular Features

Evidence suggests that young BC differs clinically from that in older patients and may be considered a distinct biological entity [31]. Young BC is typically associated with worse prognosis and unfavourable characteristics [32]. Reasons for this remain poorly understood. Women younger than 40 have increased rates of triple‐negative disease and a lower incidence of HR + BC. Despite this, HR+ disease still represents the most common subtype, accounting for two‐thirds of cases of BC in women younger than 40 years [33, 34]. This is most commonly associated with concordant HER2 positivity, with incidence of the Luminal B subtype (HR+ HER2+) over‐represented in younger women [35]. Young women are less likely to have Luminal A tumours, with percentages approximately half of those of older patients [36]. In an analysis of Luminal B BC in women < 40 compared to those > 40, younger patients had less favourable histopathological features. Young age was associated with higher tumour grade, poor differentiation and increased rates of lymphovascular invasion and nodal involvement [37].

The UK‐based POSH study demonstrated that in those age < 40 years, nodal disease was more commonly seen in ER+ BC (54%) compared to ER− tumours (42.7%) [38]. ER positivity was also associated with larger tumour size (27 vs. 26 mm), multifocal tumours (31.8% vs. 17.7%) and increased rates of lobular carcinoma (6.5% vs. 0.7%).

BC commonly metastasises to the brain, bone, liver and lung. Population‐based studies have shown that whilst those age < 50 years with TNBC were prone to development of brain metastases, those with HR+ disease developed bone metastases (bone metastases 82% in HR+ HER2− vs. 52.6% in TNBC) [39]. This pattern is typical for ER+ disease of all ages.

## 5 Current Management

1. Endocrine therapy
  Women with HR+ disease benefit from addition of hormonal agents potentially paired with suppression of ovarian function (OFS) or ovarian ablation. In pre‐menopausal women, tamoxifen is the most common endocrine therapy. Whilst aromatase inhibitors were originally reserved for post‐menopausal patients, more recently they are being used coupled with OFS in premenopausal women.
  Benefit of extending tamoxifen therapy from previously standard 5 to 10 years was demonstrated in the aTTom and ATLAS trials, with reduced recurrence and mortality [40, 41]. Oncological advantages of prolonged treatment must be balanced with compliance with therapy and side effects such as endometrial carcinoma [42, 43] and thromboembolic events [44]. It is estimated that extending therapy would increase risk of endometrial carcinoma by 2% at 15 years [45].
  Addition of OFS has proven beneficial in young women due to their increased incidence of restoration of OFS following chemotherapy. This is most commonly achieved with gonadotrophin‐releasing hormone agents. Surgical OFS can also be carried out via bilateral oophorectomy, however, this is often unsatisfactory in younger patients in whom future family planning is desired.
  Multiphase trials such as TEXT, SOFT and ASTRAA have demonstrated the benefit of addition of OFS to adjuvant endocrine therapy in pre‐menopausal women, revealing improvements in RFS, DFS and OS when compared to endocrine therapy alone [46, 47, 48]. Based on the TEXT and SOFT trials and in line with existing guidelines [49], tamoxifen alone remains the standard of care in young women with a low risk of relapse, whilst those with pre‐determined higher risk features may benefit from the addition of OFS [50]. Addition of OFS in SOFT/TEXT was associated with a greater incidence of adverse effects following commencement of treatment [51, 52]. The higher side effect rate associated with OFS risks reduced compliance.
  Because young women are at higher risk of recurrence and death from BC [53], optimising adherence is imperative for improving outcomes in this population. Younger age is associated with lower adherence to adjuvant endocrine therapy [54], with approximately one‐third of women age < 40 years either failing to commence, or prematurely discontinuing endocrine therapy [55]. This may be a contributing factor to the overall inferior survival in this group [56, 57].
2. CDK4/6 Inhibitors
  Cyclin‐dependent kinase (CDK) 4 and 6 inhibitors are now being increasingly utilised in combination with endocrine therapy in ER+ BC patients regardless of menopausal status because of the clinically significant increases in progression‐free survival and OS, as demonstrated by studies such as the MONALEESA, MONARCH and PALOMA trials [58, 59, 60, 61].
3. Chemotherapy
  Development of prognostic genomic tools such as Oncotype DX and MammaPrint have aimed to improve risk stratification in HR+ HER2− BC. The aim is to reserve chemotherapy for those who are likely to derive benefit. Studies such as RxPONDER, MINDACT and TAILORx have demonstrated that pre‐menopausal womenwomen with HR+ HER2− disease are more likely to benefit from addition of chemotherapy to standard endocrine therapy than their older counterparts.
  The RxPONDER trial evaluated the role of the recurrence score in predicting the benefit of adjuvant chemotherapy in patients with LN+ disease. Among premenopausal women, the 5‐year invasive DFS was 93.9% in those receiving chemoendocrine therapy compared to 89% for endocrine therapy alone. In post‐menopausal women, the addition of chemotherapy did not improve survival [62].
  A similar benefit was displayed in the recently updated MINDACT trial findings. This included women with HR+ HER2− BC with 0–3 positive axillary nodes and no distant metastases. In women age > 50 years, no benefit (on distant‐metastasis‐free survival) was seen from addition of chemotherapy at 8‐year follow‐up (90.2% with chemotherapy vs. 90% with endocrine therapy alone). Women age < 50 years who received chemoendocrine therapy showed improvement in distant metastases‐free survival compared to those who received endocrine therapy alone (93.6% vs. 88.6%) [63].
4. Surgery
  Studies have demonstrated an increase in the number of young women opting for mastectomy, particularly, contralateral prophylactic mastectomy, in recent years compared to breast‐conserving therapy (BCT) [64, 65]. Notably, however, in cases of stage 1 BC, BCT has been associated with a significantly higher 10‐year survival rate compared to both unilateral (HR 2.36, *p* < 0.001) and bilateral (HR 2.30, *p* < 0.001) mastectomy [66]. Furthermore, for patients with stages 1–3 disease, neither unilateral or bilateral mastectomy has shown a significant improvement in DFS over BCT [67]. While the decision to undergo bilateral mastectomy is often influenced by patient concerns about recurrence and the risk of contralateral disease [68], risk of contralateral BC remains low (0.25%–1.25% annually) [69], with further reductions following adjuvant therapy.

## 6 Specific Challenges in Young Women

1. Fertility
  The consequences of anticancer treatments including premature menopause and infertility have a significant effect on young women, both medically and psycho‐socially. The Young Women's Breast Cancer Study revealed that amongst women age < 40 years with a diagnosis of BC, 37% reported that before diagnosis they had desired future pregnancy. 51% of women expressed concerns about infertility following the completion of treatment [70].
  OFS can be altered following anticancer therapies. While menstrual function may return following cessation of treatment, premature ovarian insufficiency and infertility can persist [71]. Alkylating agents such as cyclophosphamide are associated with the greatest risk of future infertility, due to their effect on growing follicles and oocytes [72].
  While chemotherapy‐induced premature ovarian failure (POF) can be associated with improved survival outcomes [73], with larger benefit in women with HR+ disease [74, 75], it carries a significant side effect burden for young women. For women wishing to preserve fertility during chemotherapy, embryo and oocyte cryopreservation are first‐line options [76, 77]. In recent years, LHRH/GnRH agonists have been shown to preserve OFS [78]. ESMO guidelines now recommend that a GnRHa should be offered with chemotherapy to reduce risk of POF [79]. However, as data demonstrating fertility outcomes following temporary OFS is still lacking [80], it is suggested that GnRHa should not be used as replacement for established fertility preservation methods such as oocyte cryopreservation.
  Women wishing to conceive after HR+ BC are also faced with the issue of interruption of endocrine therapy. Whilst standard recommended duration of endocrine therapy is 5 years, this must be paused or ceased entirely in those planning conception. Recent data published by the POSITIVE trial collaborative group showed that interrupting endocrine therapy for pregnancy did not confer a greater short‐term risk of BC events such as distant recurrence than in the control group (8.9% vs. 9.2%) [81].
2. Bone health
  Evidence strongly suggests a significant impact of anticancer therapy in young HR+ BC on bone health. Small group studies have reported cancer‐related bone loss rates of 3%–7% in the lumbar spine and 2%–4% in the hip in premenopausal women undergoing chemotherapy [82, 83]. The negative effect on bone comes both directly as a result of therapies, and as a consequence of chemotherapy‐induced menopause. Women who undergo premature menopause display a bone mineral density (BMD) up to 14% lower than those who resume menstruation following therapy [84]. Tamoxifen has been shown to have bone‐protecting effects in postmenopausal women [85]. In contrast, however, a reduction in BMD in pre‐menopausal women has been observed [86].
  Antiresorptive therapies such as bisphosphonates may slow or prevent bone loss in these patients. Intravenous therapy is preferable to oral due to the associated gastrointestinal upset observed with oral therapy. In a BMD substudy of the ABCSG‐12, adding zolendronic acid to adjuvant endocrine therapy in premenopausal women with HR+ BC maintained BMD over 3 years, compared to a significant loss of BMD in those who did not receive zolendronic acid [87]. Benefit of addition of zolendronic acid to chemotherapy was also displayed in the PROBONEII study, whereby a reduction in BMD was seen in those receiving standard therapy + placebo, and an increase in those receiving standard therapy + zolendronic acid (−6.4% vs. +3.1% in lumbar spine) [88].

## 7 Survival

Young women with BC have a higher risk of cancer‐related death than their older counterparts [89], particularly, in HR+ disease [90]. A large registry‐based study found that cancer‐specific mortality is twice as likely in those < 40 with HR+ disease compared to those aged 40–60 independent of disease characteristics or treatment [91]. This higher mortality rate may be due to more advanced disease at diagnosis, resistance to endocrine therapies [92], resumption of menstruation post‐chemotherapy [93] and distinct tumour biology [94, 95].

While early stage HR+ disease in young women is generally associated with inferior outcomes compared to older patients, the opposite has been observed in cases of metastatic disease. Population‐based studies have suggested that women age < 40 with HR+ disease with distant metastases show significantly improved survival compared to those > 40 years [96].

ER+ tumours with high degree of proliferation tend to be more aggressive [97], suggesting the advantage of Ki67 measurement as a prognostic indicator. Expression of Ki67 is strongly associated with tumour cell growth and proliferation, with higher levels of Ki67 linked to worse prognoses [98, 99]. It was demonstrated by a Korean group that Ki67 levels were significantly higher in women with HR + BC age < 40 than those > 40 years. This was reflected in patient outcomes, with younger age and high Ki67 (> 10%) concluded to be independent predictors of poor recurrence‐free survival (RFS) [100].

## 8 Conclusion

Although the prevalence of HR+ BC is lower among young women compared to their older counterparts, HR+ disease still accounts for over 70% of BC cases in women age under 50 [6]. The incidence of this subtype has shown an upward trend in recent decades, the aetiology of which remains unclear. To refine diagnostic and therapeutic strategies for this population, there is imperative need for targeted clinical trials focusing on young women with HR+ BC, as well as in‐depth subgroup analyses within larger studies. Moreover, the increasing adoption of multi‐panel gene testing may offer significant prognostic and therapeutic advantages for younger women in the future and warrants further exploration.

## Conflicts of Interest

The authors declare no conflicts of interest.

## Synopsis

The incidence of hormone receptor‐positive breast cancer in young women has been increasing, with evidence showing significant differences compared to older women. This review summarises the current literature on HR+ breast cancer in younger patients.

## Data Availability

Data sharing is not applicable to this article as no new data were created or analysed in this study.
